# Short term effect of intravitreal bevacizumab for diabetic macular edema associated with epiretinal membrane

**Published:** 2018

**Authors:** Ashraf Khorasani Maryam, Mohammadi Tafgeh, Motallebi Mahmoud, Anvari Pasha, Sedaghat Ahad, Ghasemi Falavarjani Khalil

**Affiliations:** * Eye Research Center, Rassoul Akram Hospital, Iran University of Medical Sciences, Tehran, Iran

**Keywords:** diabetic macular edema, epiretinal membrane, Bevacizumab

## Abstract

**Purpose:** To evaluate the effect of intravitreal bevacizumab (IVB) injection on central macular thickness (CMT) in patients with diabetic macular edema (DME) associated with epiretinal membrane (ERM) and compare the results to those with DME without ERM.

**Methods:** 54 eyes of 54 patients with DME were included in this prospective comparative case series. Twenty-eight patients had ERM. All patients received 2.5 mg/ 0.1 ml IVB. The primary outcome measure was the change in central macular thickness (CMT) and the secondary outcome measure was the change in best corrected visual acuity (BCVA), one month after the IVB injection.

**Results:** All the patients completed 1-month follow-up. One month after the IVB injection, there was no statistically significant reduction in terms of CMT for the ERM group (22.64 ± 70.1 μm; P = 0.099), unlike eyes with DME alone (60.34 ± 88.5 µm; P = 0.002). Patients with ERM had a -0.09 ± 0.14 log MAR improvement in their BCVA (P =0.001) vs. 0.03 ± Log MAR change in the patients who did not have ERM (P = 0.37).

**Conclusion:** In this study, intravitreal bevacizumab resulted in improvement in BCVA in patients who had DME associated with ERM. However, in patients who only had DME, despite a reduction in CMT, no improvement in BCVA occurred. Future randomized clinical trials are warranted to precisely assess the effect of bevacizumab on the ERM.

## Introduction 

Diabetic macular edema (DME) is the most common cause of visual impairment in diabetic patients [**1**]. The estimated prevalence of DME ranged from 3.15% to 19.96% in patients with diabetes, but the prevalence of this disorder was notably variable according to the population and method of the study [**2**].

According to the ETDR study, macular laser photocoagulation was considered the standard treatment for eyes with clinically significant DME for many years [**3**]. After the introduction of anti-vascular endothelial growth factor (anti-VEGFs) drugs, many studies have shown superiority of these agents to macular photocoagulation in DME. Currently, the anti-VEGF medications are standard treatment for center-involving DME [**4**,**5**]. 

Epiretinal membrane (ERM) is a fibrocellular membranous structure over the retinal surface that presents as a hyperreflective membrane over the innermost layer of the retina in optical coherence tomography (OCT) [**6**]. Diabetic retinopathy is one of the most important risk factor for ERM formation. ERM has been reported in 14-30% of the eyes with center involving diabetic macular edema [**7**,**8**]. In addition, eyes with DME who received intravitreal injection of anti-VEGFs may be at risk of ERM formation [**9**]. 

The simultaneous presence of ERM and diabetic macular edema may affect the therapeutic role of anti-VEGF agents on DME. To date, only very few studies have focused on effect of epiretinal membrane on outcomes of intravitreal anti-VEGFs for treating DME and results of these studies are not consistent [**10**,**11**]. Also, none of them has been specifically studied the effect of Bevacizumab. The aim of this study was to evaluate the effect of ERM on anatomical and visual outcomes of intravitreal Bevacizumab injection (IVB) in eyes with center involving DME.

## Methods 

Consecutive patients with DME with and without ERM were included in this prospective comparative case series, from October 2014 to October 2017. The study protocol was approved by the Institutional Review Board/ Ethics Committee of the Eye Research Center of Rasoul Akram Hospital and carried out in accordance with the Helsinki Declaration. The patients included had to have DME with a central macular thickness more than 300 microns and best corrected visual acuity (BCVA) between 20/ 400 and 20/ 40. Patients with proliferative diabetic retinopathy, any ocular disease other than diabetic retinopathy (including glaucoma, cataract, and uveitis), any intravitreal injection (anti-VEGF, steroid) within the past three months, previous vitreoretinal surgery, any ocular surgery (except for uncomplicated phacoemulsification within the past six months), and history of macular photocoagulation in past three months were excluded. Informed consents were obtained. 

Complete ophthalmic examination (BCVA measurement with E chart, slit lamp biomicroscopy, fundus examination, and Goldmann applanation tonometry) and retinal imaging using spectral domain OCT (Spectralis; Heidelberg Engineering, Heidelberg, Germany) was performed for all patients before and one month after IVB. 

OCT scans were obtained for all patients, using either Spectralis HRA-OCT (Heidelberg Engineering, Heidelberg, Germany) or Topcon OCT (1000-type 2, Tokyo, Japan) instruments. The OCT device was the same for each patient before and after injections. The OCT volume scan size was 20°×20° area for Spectralis, and 6 × 6 mm² 3D scan pattern for Topcon device. Average thickness of points within the 1-mm circle of the center of fovea in each OCT device was recorded as CMT. Epiretinal membrane was defined as a hyper-reflective layer over the retinal surface. Eyes with vitreomacular traction were excluded. 

Intravitreal bevacizumab (Avastin; Genentech Inc, South San Francisco, CA) was performed for all patients under sterile condition in operating room. After applying topical tetracaine 1%, periocular skin and eyelid and eyelashes were scrubbed with povidone-iodine 10%, then eyelid speculum was inserted and ocular surface and fornices were rinsed with 1-2 drops of povidone-iodine 5% for 2 minutes. Intravitreal injection of 2.5 mg (0.1 ml) bevacizumab was administrated using a 30-gauge needle.

The main outcome measure was changes in central macular thickness (CMT), one month after injection. The secondary outcome measure was the change in BCVA. 

For statistical analysis, Snellen visual acuity was converted to logarithm of the minimum angle of resolution (log MAR). Changes in BCVA and CMT were compared between the two groups. Data were analyzed using SPSS software version 16 (SPSS Inc., Chicago, IL, USA). Variables were checked for normal distribution with the Kolmogorov–Smirnov test. Mann–Whittney U test was used to make a comparison between the groups and the Wilcoxon test was used to analyze changes in the variables before and after injection. A P value less than 0.05 was considered statistically significant.

## Results 

Twenty-eight eyes of 28 patients (12 males and 16 females) with ERM and twenty-six eyes of 26 patients (12 males and 1 female) without ERM were enrolled into the study. Baseline characteristics are presented in **Table 1**. The age, sex, lens status, duration of DM and the presence of hypertension were statistically similar between the groups. 

**Table 1:** Baseline characteristics of patients included in the study

	DME without ERM	DME with ERM	P value
No. of eyes (patients)	26(26)	28(28)	
Eye (right/ left)	18/ 8	18/ 8 14/ 14	0.151
Average age (±SD), years	59.9 ± 9.7	60.7 ± 8.0	0.518
Sex (male/ female)	12/14	12/16	0.651
Duration of DM (years ± SD)	15.2 ± 4.4	13.5 ± 4.7	0.806
Hypertension (yes/ no)	9/ 17	11/ 17	0.723
Lens status (phakic/ pseudophakic)	23/ 3	19/ 9	0.069
Average BCVA at baseline (log MAR ± SD)	0.57 ± 0.26	0.88 ± 0.29	0.000
Average CMT at baseline (micron ± SD )	446.96 ± 125.48	479.28 ± 110.43	0.319
Average BCVA at one month (log MAR ± SD)	0.60 ± 0.33	0.78 ± 0.26	0.035
Average CMT at one month (micron ± SD )	386.61 ± 114.73	456.64 ± 123.54	0.036
ERM = Epiretinal membrane, BCVA = Best corrected visual aculity, CMT = Central macular thickness, DME = Diabetic macular edema, Log MAR = Logarithm of minimal angle of resolution.			

Baseline central macular thickness was statistically similar between the two groups; however, baseline BCVA in DME associated with ERM was statistically significantly worse than in the no-ERM group (0.88 ± 0.29 versus 0.57 ± 0.26, P < 0.001).

One month after the injections, the mean CMT was 456.6 ± 123.5 µm in the ERM group and 386.6 ± 114.7 µm in the no-ERM group (P=0.036). The statistically significant decrease in CMT (P=0.002) was detected in the no-ERM group, but not in the ERM group (P=0.09).

One month after the injection, the mean BCVA in the ERM group was 0.78 ± 0.26 logMAR, and in the no-ERM group was 0.60 ± 0.33 logMAR (P=0.35). The BCVA change was statistically significant in the ERM group, but not in the no-ERM group (P=0.002 and P=0.324, respectively). The mean change in BCVA was -0.09±0.14 logMAR in the ERM group and 0.03± 0.16 logMAR in the no-ERM group (P=0.004). 

## Discussion 

DME is one of the most common causes of blindness in diabetic patients [**1**]. Currently, intravitreal injection of anti-VEGFs drugs is the most effective and safe treatment for DME causing decreased vision [**12**,**13**]. Approximately 30% of the eyes with DME may have ERMs [**14**]. Few studies have investigated the effect of ERM on the therapeutic effects of anti-VEGFs agents on DME [**10**,**11**] and there is no study reported the impact of ERM specifically on bevacizumab effects for treating DME. Therefore, this is the first report specifically describing the effects of IVB on DME associated with ERM.

Several studies have reported the effect of ERM on response to anti-VEGF agents in retinochoroidal diseases other than diabetic retinopathy [**15**-**19**]. In a retrospective chart review of 63 eyes of 63 patients, Alkin et al. did not find any significant differences in terms of BCVA improvement, central retinal thickness (CRT) change and mean number of bevacizumab injections in eyes with naive treatment newly diagnosed neovascular AMD alone, and those associated with ERM in an over 24-months follow-up [**15**]. Chatziralli et al. stated that in a one-year follow-up, patients with wet AMD and ERM needed more injections than patients with wet AMD alone. Both groups had significant BCVA improvement and CRT reduction, although there was no significant difference in BCVA and CRT between the two groups at the end of the follow-up [**16**]. Cho et al. concluded that the presence of ERM in eyes with neovascular AMD decreases the effect of anti VEGFs intravitreal injection in reducing central foveal thickness but does not have an effect on visual improvement by using this drug [**17**].

In this study, we found significant improvement in BCVA despite the insignificant reduction in CMT. In the no-ERM group, the BCVA remained unchanged despite significant reduction in CMT. Baseline BCVA was different between the two groups; however, the main outcome measure was CMT, which was similar at baseline. Yoon et al. noted that the presence of vitreomacular interface abnormalities reduced the effect of anti-VEGFs (ranibizumab and bevacizumab) on DME. Their study included ERM associated with DME as a vitreomacular interface abnormality. In contrast with our findings, baseline BCVA did not differ between groups, and after three injections both groups showing significant improvement compared to baseline BCVA but BCVA increase was statistically significant higher in the DME group. This finding can be related to the inclusion criteria that in their study any abnormalities in vitreomacular interface were included [**10**]. Ercalik and his associates found that intravitreal ranibizumab in patients with DME and ERM reduced CMT significantly but the change in BCVA after injection was not significant, both of BCVA and CMT reduced significantly in patients with DME alone. Significantly worse baseline BCVA in their study was consistent with our study. They hypothesized that insignificant improvement in BCVA was associated with the worse baseline BCVA in DME/ ERM group and this difference was attributable to the older age of this group [**11**].

Several studies have reported the absence of a linear correlation between the improvement in BCVA and the reduction of CMT [**20**-**22**]. The contradiction between the various studies that examined the effect of ERM on the response to treatment with anti-VEGFs, intravitreal triamcinolone, or macular photocoagulation may be due to the fact that there are other parameters in the OCT that can predict the BCVA in DME more strongly than the retinal thickness [**23**-**27**]. Disruption of the photoreceptor IS/ OS junction and the integrity of the external limiting membrane are other important parameters for BCVA prediction in patients with DME [**23**,**25**-**27**]. 

Our study had several limitations. The sample size was small, the follow up was short, and the groups were not matched for BCVA. Also, the pattern of diabetic macular edema, and the integrity of inner and outer retinal layers were not analyzed. The response to treatment may be different based on the pattern of DME [**11**,**28**].

In conclusion, this study demonstrated that after the single injection of intravitreal Bevacizumab, BCVA improved significantly in eyes with DME associated with ERM and resulted in significant CMT reduction in eyes with DME alone. Future larger studies are needed to determine the effect of ERM on the response to anti-VEGF agents. 

**Financial interest**

None of the authors has any financial interest in the subject matter of this paper. 

**Acknowledgement**

Authors would like to thank Dr. S. Bakhti for her contribution in gathering the data of some patients.
